# Ischiopagus Tripus Conjoined Twins

**Published:** 2011-03-10

**Authors:** Yousuf A. Khan

**Affiliations:** Department of Pediatric Surgery, National Institute of Child Health Karachi, Pakistan

**Keywords:** Conjoined Twins, Ischiopagus, Tripus, Tetrapus

## Abstract

A conjoined twin is one of the rare congenital defects. Ischiopagus variety is even rarer. We present a case of ischiopagus-tripus conjoined twins. They were fused at the lower halves of the bodies. One of the twins was apparently normal looking, active and pink. The other twin was small, ill looking, sluggish and cyanosed. There were two well formed separate lower limbs on one side and a fused limb at the other side. The twins had an imperforate anus and two small orifices draining urine with incompletely developed external genitalia. Pre-operative workup was in progress when the twins passed away.

## INTRODUCTION

Conjoined twins represent one of the rarest congenital anomalies occurring with a varying incidence of about 1:50,000-1:200,000 births approximately. Females predominate over males in the order of 2:1 to 3:1. Ischiopagus conjoined twinning is even rarer representing only 6% of all conjoined twins. Because of the highly variable and complex anatomy and associated malformations, skilled clinical assessment aided by detailed radiological studies, appropriate planning and team work are required for the successful separation of the conjoined twins [1, 2, 3, 4]. We present here a case of ischiopagus tripus conjoined twins who passed away while being investigated.

## CASE REPORT

Full term conjoined twins, weighing 4000 grams, were brought to our hospital at 3rd hour of life. They were delivered by emergency cesarean section, to a 32 years old lady, already having 8 children. There was no family history of twinning. Ante-natal ultrasound was done only once 4 days prior to delivery. It showed twin alive pregnancy of 38 weeks ± 01 week with cephalic presentation and adequate liquor with no comments on conjoining.

The twins were fused at the lower halves of their bodies with a single umbilicus at the mid. There was a horizontal scar of about 3 ½ cm extending to the right of umbilicus. The healthier looking active pink neonate, termed Twin A, had normal head, neck and upper limbs. There was gradual broadening of the torso above umbilicus. The smaller neonate, termed Twin B, was a thin, emaciated, cyanosed, microcephalic neonate, with small torso and feeble reflexes. There were two separate lower limbs at the left side of Twin A and a fused lower limb at the right side. The anal orifice was absent and two orifices were found in perineum draining urine. Umbilicus had a single set of umbilical vessels. External genitalia were incompletely developed to assign gender to either neonate. Cardiac murmur was audible on auscultation in both the twins (Fig. 1, 2).

**Figure:**
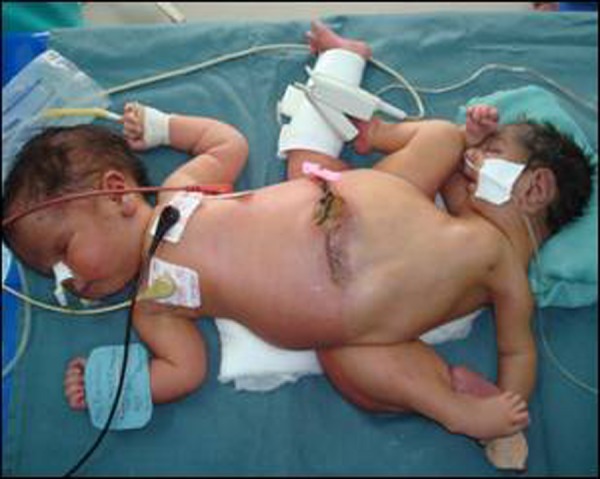
Figure 1: Ischiopagus tripus conjoined twins

**Figure:**
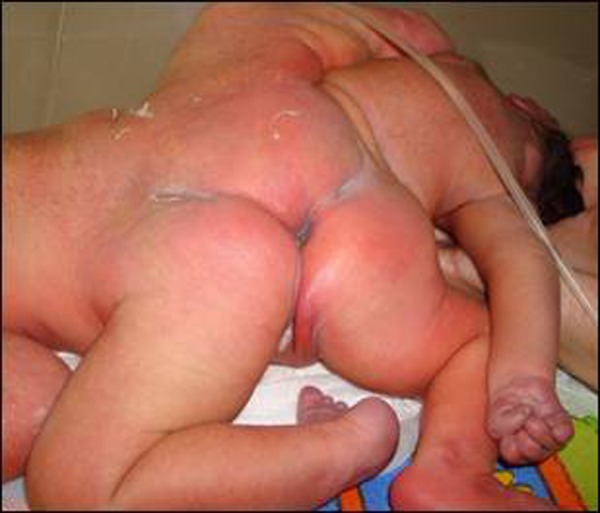
Figure 2: Posterior view

X-ray babygram revealed fusion at the lower abdomino-pelvic region. Scoliosis of dorsolumbar spine noted in both neonates, which was more marked in the Twin B. Pubic symphyses were widely separated and ischio-pubic bones were deformed on the right side with sacral dysgenesis of Twin B. The hip joints were abnormally oriented and horizontally oriented femora were attached to a large pelvic ring bilaterally. On one side, there was synostosis of femora. Calvarial defect with torticollis was present in Twin B (Fig. 3).

**Figure:**
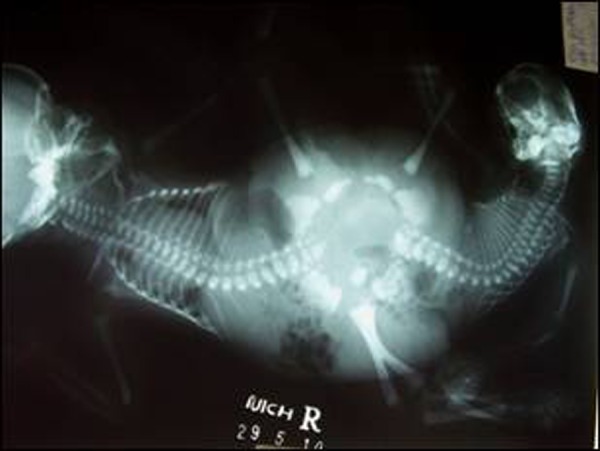
Figure 3: X-ray babygram of the conjoined twins

Ultrasound abdomen examination of Twin A showed hydronephrosis and hydroureter on both the sides while rest of the viscera were normal. In the Twin B, spleen and the left kidney were well formed but other viscera could not be clearly visualized. There was a large anechoic cystic structure in the pelvis representing bladder. Cystogram performed via the two small orifices in the perineum revealed non-visualization of urethra with pooling of contrast in the single reservoir representing shared urinary bladder. (Fig. 4)

**Figure:**
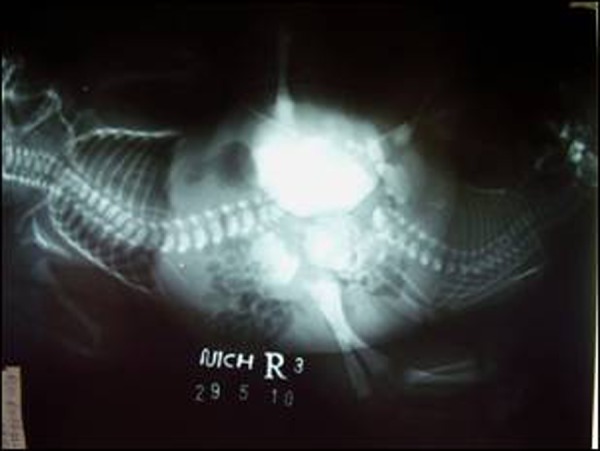
Figure 4: Cystogram - showing pooling of contrast in a single reservoir (common urinary bladder)

MRI revealed moderate hydronephrosis bilaterally in both babies. In Twin B there was lack of visualization of other viscera. Normal lung and mediastinum were also not seen in Twin B who also had small skull, deformed and devoid of normal brain tissue, replaced by a cystic space. The Twin A showed normal thoraco-abdominal viscera bilaterally. Multiple prominent cystic areas were noted in the centre likely representing bowel loops. There was failure to identify separate urinary bladders. Because of the limited multi-dimensional views of MRI, internal genitalia were not appreciable. (Fig. 5)

**Figure:**
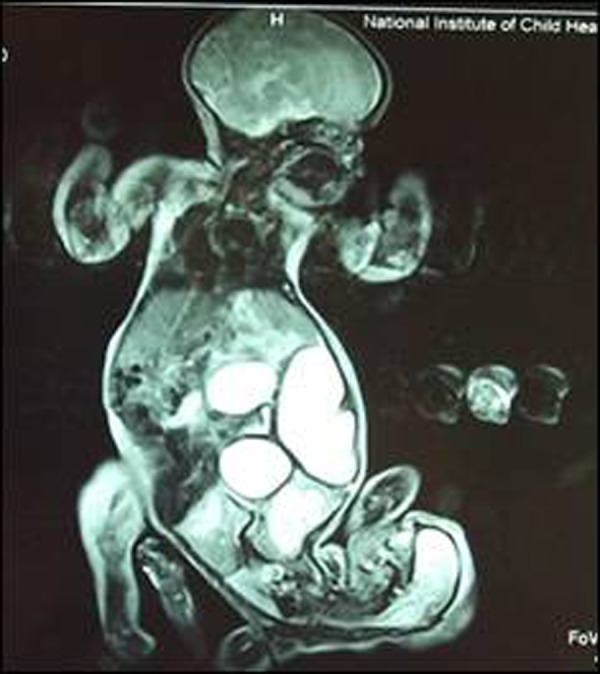
Figure 5: MRI of conjoined twins

On the second day of life, sigmoid loop colostomy was made under local anesthesia through the already present scar on the abdomen. The peritoneal cavity was not explored. The stoma started functioning following day and the Twin A was allowed top feed via nasogastric tube that was tolerated. The clinical condition of the Twin B remained critical throughout with feeble reflexes, abnormal breathing patterns and oxygen desaturation most of the times. Twin B who later died following which Twin A’s condition also deteriorated but he also passed away within few hours on the 6th post-operative day. 

## DISCUSSION

 Conjoined twin is a rare developmental defect; the exact cause of which is still obscure. Some authorities consider it as a result of incomplete division of the embryonic disc, while others are of the view that there is secondary fusion of two originally separate monozygotic embryonic discs [5].

Various classification systems have been proposed for this defect. Spencer classified conjoined twins on the basis of site of union i.e.: ventral or anterior union (cephalopagus, thoracopagus, omphalopagus, ischiopagus, and parapagus) and dorsal or posterior union (craniopagus, pyopagus, and rachipagus), while Potter and Craig simply classified on the basis of most common forms of twinning [6, 7].

Ischiopagus conjoined twinning considered the most complicated form, accounts only for 6% of the cases. Grossly, ischiopagus usually lie along a long axis with heads on opposite sides. They have a common umbilicus and the bodies fuse below this level, sharing lower abdomen and pelvis. About half of the ischiopagus have four separate lower limbs, 1/3rd have 3 lower limbs (2 separate and 1 fused) attached to the body laterally and 1/5th cases are parasitic [1, 2, 3, 4]. Our case, therefore, is an example of ischiopagus tripus variety. Radiologically, there was synostosis of the femora on the right side of Twin A. (Fig. 2). It is reported that one of the twins is almost always weaker or smaller, though genetically identical and may have additional congenital defects [5]. This was also noted in this case as the Twin B was very small, weak and microcephalic with scoliosis of dorsolumbar spine and sacral dysgenesis. Calvarial defect, torticollis and absence of normal brain tissue were also present.

The internal organs shared by ischiopagus conjoined twins are usually the liver, lower gastrointestinal tract (GIT) and genitor-urinary organs. The GIT sharing in ischiopagus twins is usually ileo-colic, having union at the terminal ileum (at Meckel’s diverticulum) with shared bowel distally or rarely the sharing is only colonic. The rectum and the anus may be single or imperforated. Obstructed GI or genitourinary tract, ruptured omphalocele, severe respiratory distress or cardiac failure in one of the twins, are some of the indications which may require emergency surgery [1, 2, 5, 8].

It is well known fact that the fetal wounds heal without scar formation. The true mechanism of the scarless fetal tissues repair is not known [9]. The linear healing wound or scar at the right side of umbilicus in our patient could not be explained according to this theory. This scar was also the line of vascular perfusion demarcation between the twins frequently noted when Twin B used to desaturate.

About 51% of the ischiopagus twins have shared pelvic organs. Urinary bladder may be single or double, lying side by side or combined with one bladder draining into the other [5]. In this case, the twins had two perineal openings which were draining urine with single urinary bladder as noted on cystogram, ultrasound abdomen and MRI.

Diagnostic workup also includes skeletal surveys, ultrasonography, contrast imaging of the gastrointestinal and urinary systems, endoscopy, computed tomography and MRI. Angiography and radio-isotope liver and renal scans may provide with further details. Complete cardiac evaluation ought to be done in ischiopagus, as there is high incidence of associated congenital cardiac anomalies in all variants of conjoined twins [1, 3, 5]. We employed many of these investigations for complete delineation of external and internal structures, and to detect associated anomalies in order to plan separation but unfortunately twins died during the course of hospital stay.

## Footnotes

**Source of Support:** Nil

**Conflict of Interest:** None declared

## References

[1] O’Neill JA Jr, Holcomb GW III, Schnaufer L, Templeton JM Jr, Bishop HC, Ross AJ III (1988). Surgical experience with thirteen conjoined twins. Ann Surg.

[2] Sangari SK, Khatri K, Pradhan S (2001). Omphalopagus ischiopagus tetrapus conjoined twins - a case report. J Anat Soc India.

[3] Kingston CA, McHugh K, Kumaradevan J, Kiely EM, Spitz L (2001). Imaging in the preoperative assessment of conjoined twins. Radiographics.

[4] Zhang XS, Feng ZG, Xiong QX, LI MJ, Tang DX (2007). Successful separation of ischiopagus tetrapus conjoined twins. World J Pediatr.

[5] Rode  H, Fieggen  AG, Brown  RA, Cywes  S, Davies  MRQ, Hewitson  JP (2006). Four decades of conjoined twins at Red Cross Children’s Hospital - lessons learned.. S Afr Med J.

[6] Spencer  R (1996). Anatomic description of conjoined twins: A plea for standardized terminology. J Pediatr Surg.

[7]  O’Neill JA Jr (2006). Conjoined Twins. In: O’ Neill JA Jr, Rowe MI, Grosfeld JL, Fonkalsrud EW, Coran AG, editors. Pediatric Surgery.

[8] Mughal  SA,  Shoukat  M,  Soomro  S,  Shaikh  JM,  Abbasi  MP ( 2004). Ischiopagus tripus conjoined twins. A Case Report. J Surg Pak.

[9] Adzick  NS,  Longaker  MT ( 1992). Scarless Fetal healing-therapeutic implications. Ann Surg.

